# Density of Sponge Gold Filling

**Published:** 1855-07

**Authors:** A. Snowden Piggot


### *Density of Sponge Gold Filling.* By A. Snowden Piggot, M. D.

During the visit of Dr. W. H. Dwinelle, to Baltimore, last spring, I ascertained, at his instance, the specific gravity of a filling made with H. J. Watts & Co's sponge gold. It was cylindrical in form, having a slight bevel upon one side of the summit, and was finely polished.

I took the specific gravity at the temperature of maximum density of water, and found it to be 18.88, fused gold being only 19.2, and the pure beaten metal rarely going above 19.4.

It is proper to state that I was informed by Dr. Dwinelle, that in the process of condensing this filling, the point of one of the steel instruments used for that purpose broke off. This would, of course, reduce the specific gravity of the cylinder, so that it was in reality denser than my figures indicate.

